# Supplementary material for: Targeting Oxidative Phosphorylation Reverses Drug Resistance in Cancer Cells by Blocking Autophagy Recycling
Source: Cells. 2020 Sep 1;9(9):2013. doi: 10.3390/cells9092013 (PMC7565066; doi:10.3390/cells9092013)
Supplement: Supplementary file 1 [file cells-09-02013-s001.pdf]

A

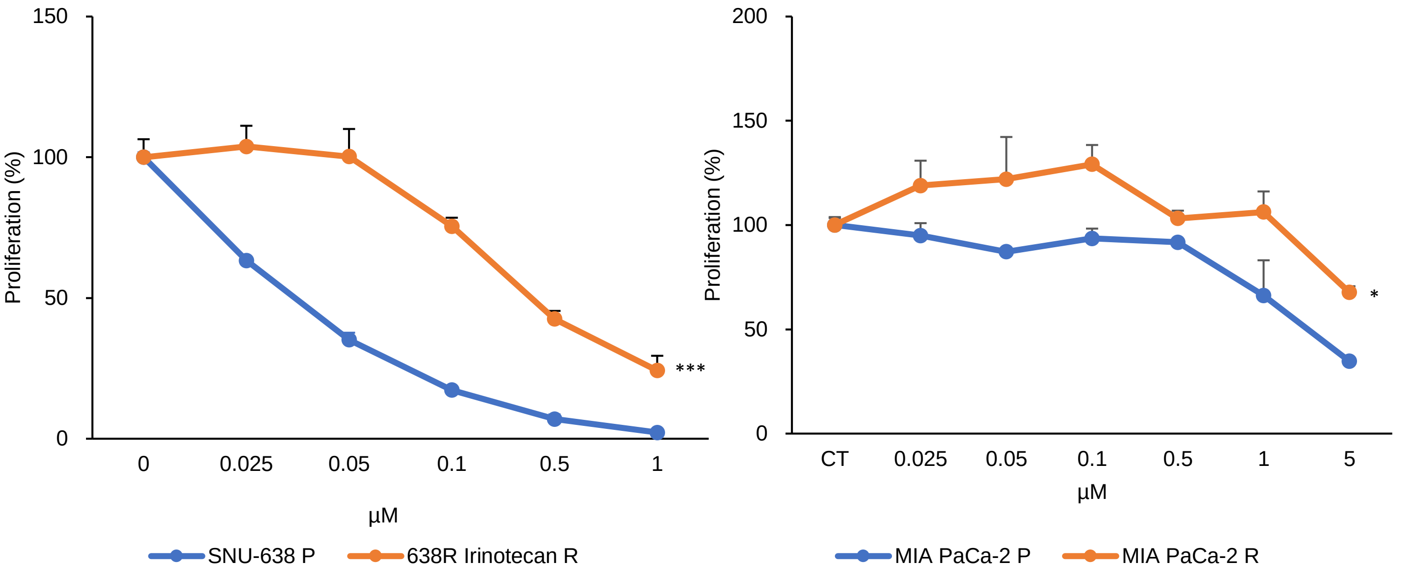

Figure S1. Irinotecan-resistant cancer cells were established through long-term treatment.
The effect of irinotecan on irinotecan-resistant SNU-638 and MIA PaCa-2 cells after 48 h was determined by SRB assay (n=3). Each bar represents the mean + s.d. *p < 0.05, **p < 0.01, ***p < 0.001.

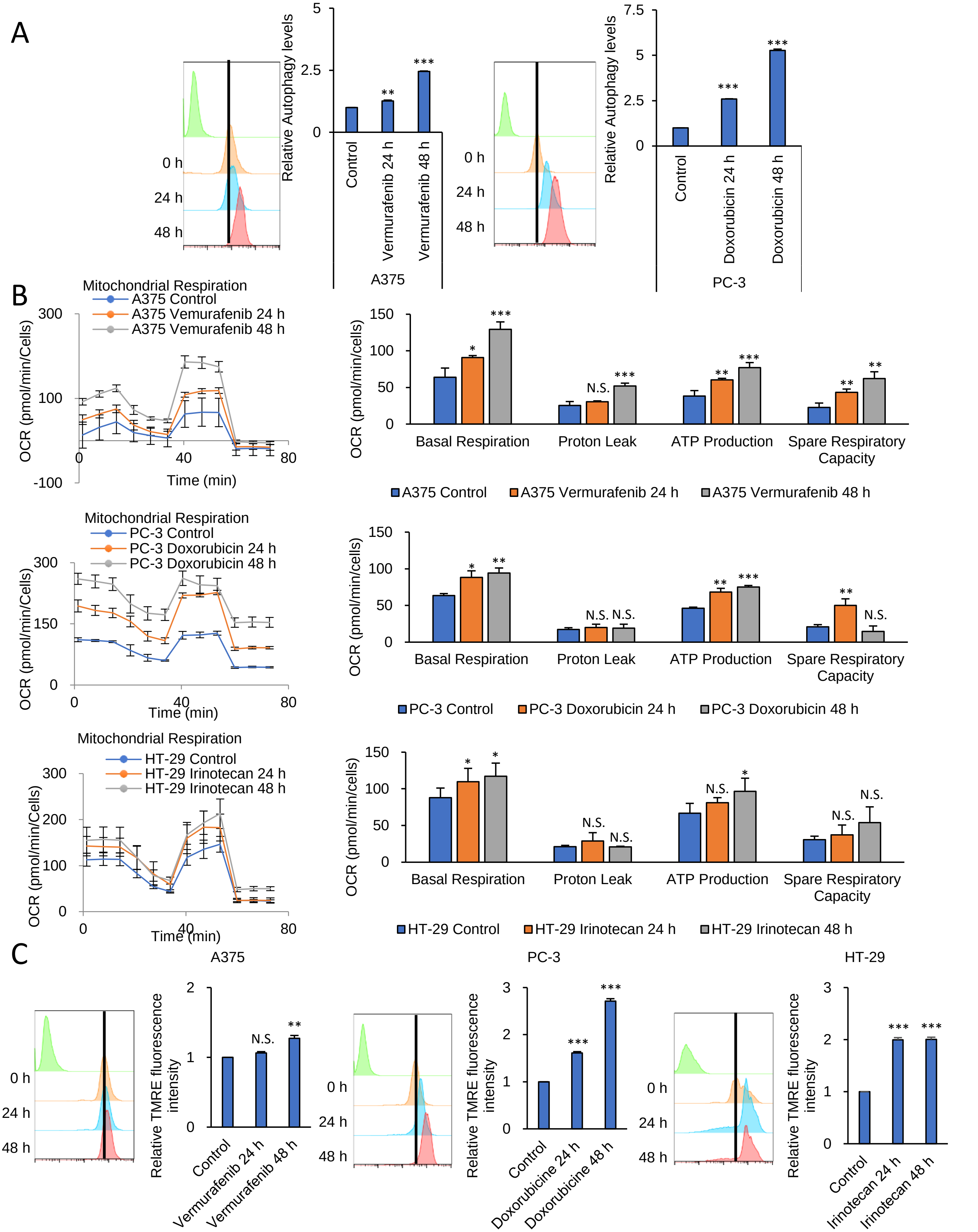

Figure S2. Anti-cancer drug treatment induces autophagy and Oxygen Consumption Rate.
(A) Autophagy levels were analyzed by Cyto-ID autophagy detection kit in anti-cancer drug treatment cancer cells and non-treatment cancer cells (Vermurafenib; 0.5 µM and Doxorubicin; 1 µM treatment). (B) Oxygen Consumption Rates and respiration parameters were analyzed in A375, PC-3 and HT-29 after treatment anti-cancer drug (Vermurafenib; 0.5 µM, Doxorubicin; 1 µM and Irinotecan; 0.5 µM) treatment for 24 h and 48 h (n=4). (C) Mitochondrial membrane potential was Analyzed by staining TMRE. Mitochondrial membrane potential was increased in anti-cancer drug treatment cancer cells compared to non-treatment cancer cells (n=3). Each bar represents the mean + s.d. *p < 0.05, **p < 0.01, ***p < 0.001.

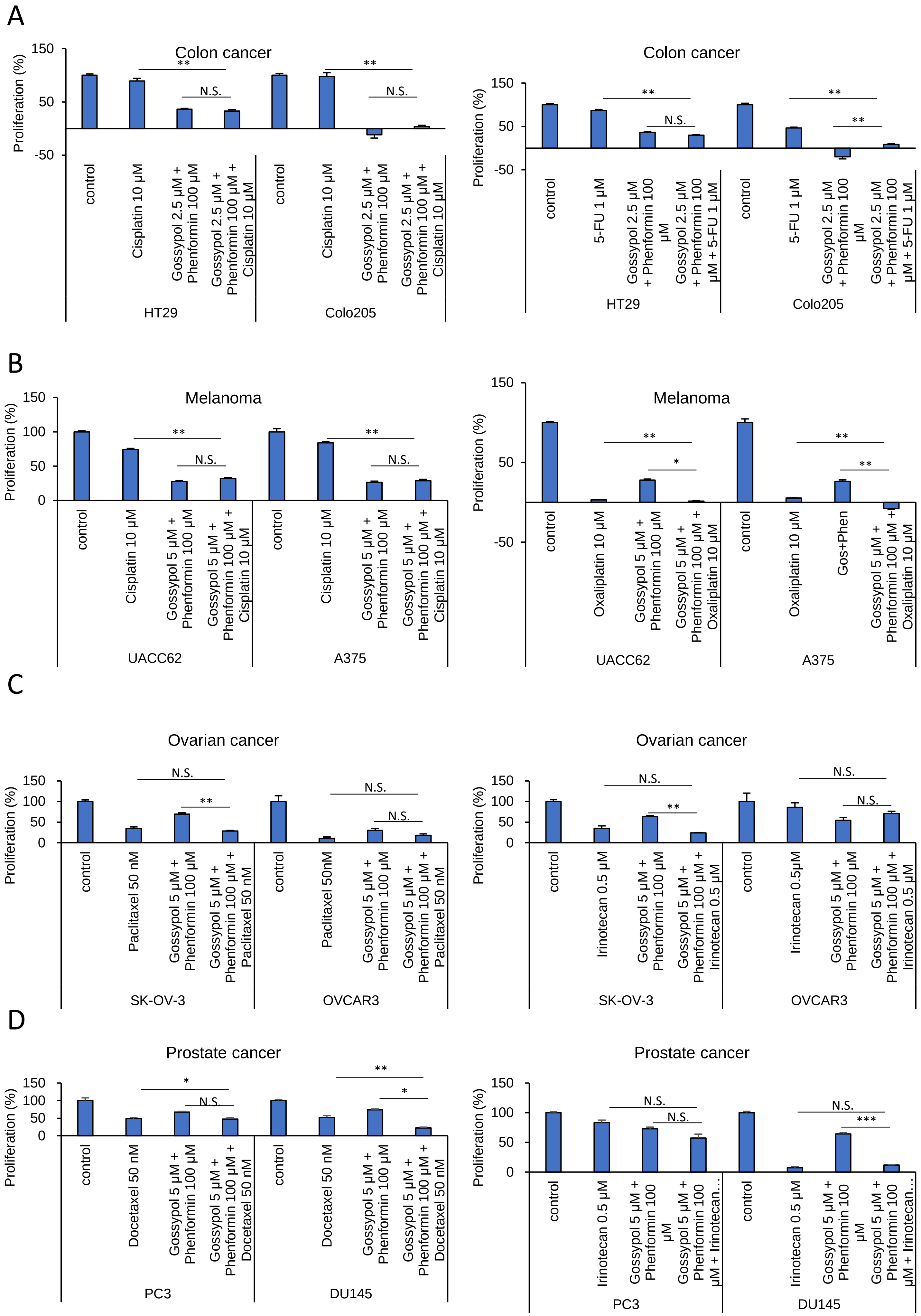
A
Colon cancer
Proliferation (%)
150
50
-50
**
N.S.
**
N.S.
control
Cisplatin 10 μM
Gossypol 2.5 μM + Phenformin 100 μM
Gossypol 2.5 μM + Phenformin 100 μM + Cisplatin 10 μM
HT29
Colo205
Colon cancer
5-FU 1 μM
Gossypol 2.5 μM + Phenformin 100 μM
Gossypol 2.5 μM + Phenformin 100 μM + 5-FU 1 μM
B
Melanoma
100
0
UACC62
A375
Oxaliplatin 10 μM
Gossypol 5 μM + Phenformin 100 μM
Gossypol 5 μM + Phenformin 100 μM + Oxaliplatin 10 μM
Gos+Phen
*
C
Ovarian cancer
Paclitaxel 50 nM
Paclitaxel 50nM
Gossypol 5 μM + Phenformin 100 μM + Paclitaxel 50 nM
SK-OV-3
OVCAR3
Irinotecan 0.5 μM
Irinotecan 0.5μM
Gossypol 5 μM + Phenformin 100 μM + Irinotecan 0.5 μM
D
Prostate cancer
Docetaxel 50 nM
Gossypol 5 μM + Phenformin 100 μM + Docetaxel 50 nM
PC3
DU145
Gossypol 5 μM + Phenformin 100 μM + Irinotecan...
***

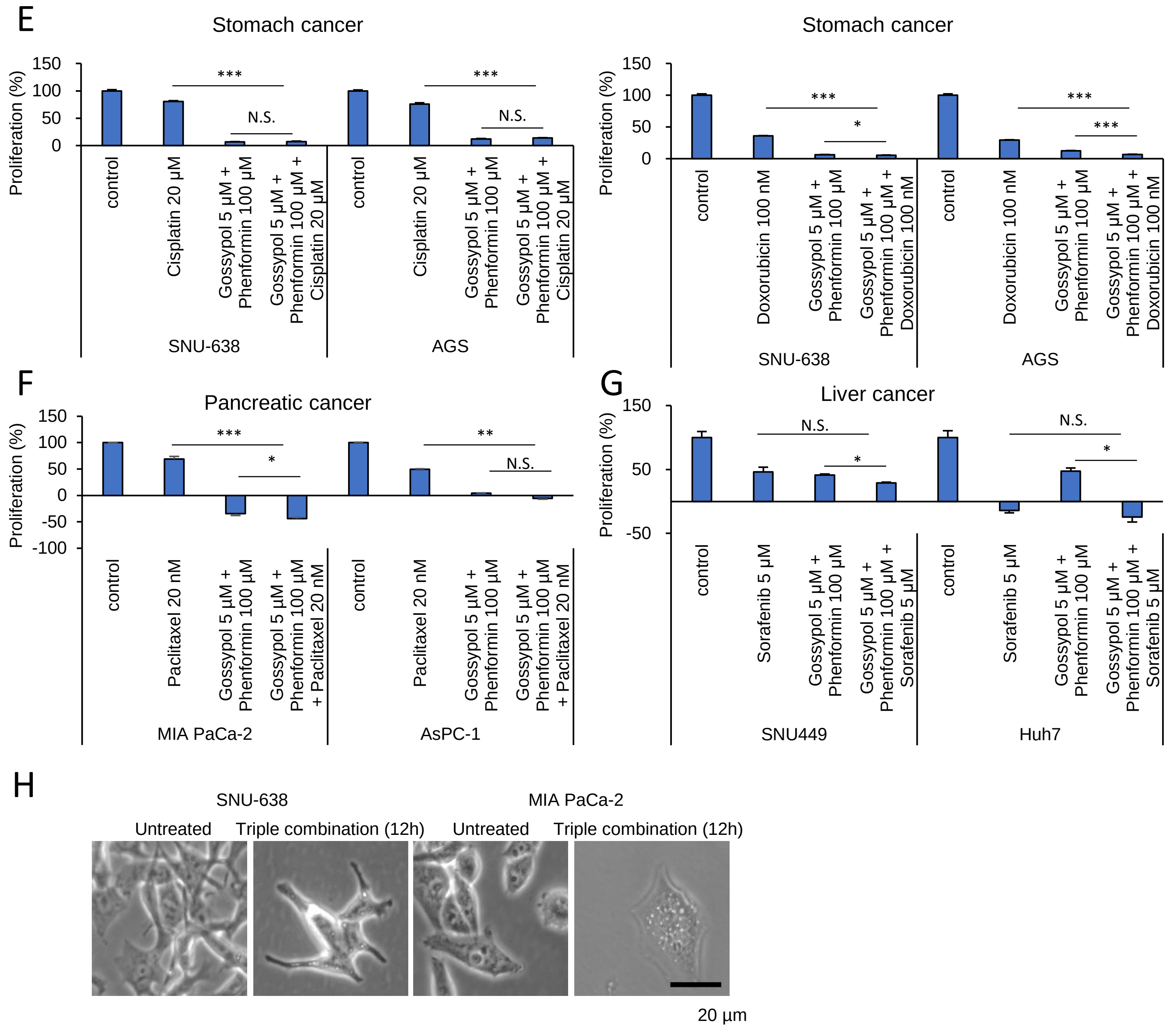

Figure S3. The cell death induced by triple combination treatment showed a Autophagic cell death morphology. (A-G) Non-synergistic effect of triple-combined treatment of 2.5 μM or 5 μM gossypol, 100 μM phenformin and indicated anti-cancer drug after 48 h on cell proliferation was determined by SRB assay. (H) MIA PaCa-2 and SNU-638 cells were treated with triple-combination of 5 μM gossypol, 100 μM phenformin and irinotecan (SNU-638; 0.5 μM and MIA PaCa-2 ; 1 μM) for indicated times, and then cells were imaged by means of a microscope. Scale bar = 20 μm.
Each bar represents the mean + s.d. *p < 0.05, **p < 0.01, ***p < 0.001.

A

Cell line	Colo 205
Mice	BALB/c-nude (Orient)
N (Head)	3
Drug delivery	Gossypol, Phenformin: PO<br>Irinotecan: IP
Treatment on/off	Gossypol, Phenformin: On (6) Off (1)<br>Irinotecan: On (1) Off (6)
Week	3
Drug dose	Gossypol: 80 mg/kg<br>Phenformin: 100 mg/kg<br>Irinotecan: 40 mg/kg
Drug vehicle	Gossypol and Phenformin: 5 % DMSO, 5 % cremophor and 90 % PBS<br>Irinotecan: 5 % DMSO and 95 % water
IRB Number	NCC-18-435

B

Cell line	A375
Mice	BALB/c-nude (Orient)
N (Head)	7
Drug delivery	Gossypol, Phenformin and Vemurafenib: PO
Treatment on/off	Gossypol, Phenformin: On (6) Off (1)<br>Vemurafenib: On (5) Off (2)
Week	3
Drug dose	Gossypol: 80 mg/kg<br>Phenformin: 100 mg/kg<br>Vemurafenib: 30 mg/kg
Drug vehicle	Gossypol and Phenformin: 5 % DMSO, 5 % cremophor and 90 % PBS<br>Vemurafenib: 4 % DMSO + 30 % PEG300 + 5 % Tween 80 + 61 % water
IRB Number	NCC-18-435

C

Cell line	SK-OV-3
Mice	BALB/c-nude (Orient)
N (Head)	4
Drug delivery	Gossypol, Phenformin: PO<br>Cisplatin: IP
Treatment on/off	Gossypol, Phenformin: On (6) Off (1)<br>Cisplatin: On (2) Off (5)
Week	6
Drug dose	Gossypol: 40 mg/kg<br>Phenformin: 100 mg/kg<br>Cisplatin: 4 mg/kg
Drug vehicle	Gossypol and Phenformin: 5 % DMSO, 5 % cremophor and 90 % PBS<br>Cisplatin: water
IRB Number	NCC-18-435

D

Cell line	PC-3
Mice	BALB/c-nude (Orient)
N (Head)	4
Drug delivery	Gossypol, Phenformin: PO<br>Doxorubicine: PO
Treatment on/off	Gossypol, Phenformin: On (6) Off (1)<br>Doxorubicin: On (5) Off (2)
Week	2
Drug dose	Gossypol: 80 mg/kg<br>Phenformin: 100 mg/kg<br>Doxorubicin: 5 mg/kg
Drug vehicle	Gossypol and Phenformin: 5 % DMSO, 5 % cremophor and 90 % PBS<br>Doxorubicin: 5 % DMSO and 95 % water
IRB Number	NCC-18-435

E

Cell line	SNU-638
Mice	BALB/c-nude (Orient)
N (Head)	5
Drug delivery	Gossypol, Phenformin: PO<br>Irinotecan: IP
Treatment on/off	Gossypol, Phenformin: On (6) Off (1)<br>Irinotecan: On (1) Off (6)
Week	5
Drug dose	Gossypol: 80 mg/kg<br>Phenformin: 100 mg/kg<br>Irinotecan: 10 mg/kg
Drug vehicle	Gossypol and Phenformin: 5 % DMSO, 5 % cremophor and 90 % PBS<br>Irinotecan: 5 % DMSO and 95 % water
IRB Number	NCC-20-558

F

Cell line	MIA PaCa-2
Mice	BALB/c-nude (Orient)
N (Head)	5
Drug delivery	Gossypol, Phenformin: PO<br>Irinotecan: IP
Treatment on/off	Gossypol, Phenformin: On (6) Off (1)<br>Irinotecan: On (1) Off (6)
Week	4
Drug dose	Gossypol: 80 mg/kg<br>Phenformin: 100 mg/kg<br>Irinotecan: 40 mg/kg
Drug vehicle	Gossypol and Phenformin: 5 % DMSO, 5 % cremophor and 90 % PBS<br>Irinotecan: 5 % DMSO and 95 % water
IRB Number	NCC-20-558

Table S1. Xenograft mouse models conditions table.
